# Eight ways to get a grip on implementing mindfulness sessions in medical schools

**DOI:** 10.36834/cmej.57011

**Published:** 2020-03-16

**Authors:** Tatiana Rac, Anita Chakravarti

**Affiliations:** 1 Medical Resident, University of British Columbia, British Columbia, Canada; 2 College of Medicine, University of Saskatchewan, Saskatchewan, Canada; 3 Saskatchewan Health Authority, Saskatchewan, Canada; 4 [M]Power Mindful Professional Practice, Saskatchewan, Canada

Many medical colleges have explored Mindfulness Practice Sessions (MPS) to help their medical students cope with the demands of training.^1^^-^^5^ In the last decade, the awareness and development of physician wellness/mindfulness has flooded scientific and popular literature. A growing body of works shows that mindfulness-based interventions can decrease stress, anxiety, and depression, as well as improve mood, self-efficacy, and empathy in health profession students.^1^^-^^5^ Since 2009, we have explored a modified experiential program of one-hour weekly practice sessions as an introductory experience for the more time and resource-intensive Mindfulness Based Stress Reduction® or Mindful Practice ® (mindfulness, narrative medicine, and appreciative inquiry).^6^^-^^10^ This article explains how best to develop sustainable mindfulness curricula in health science colleges.

We realized many potential pitfalls could be addressed ahead of time with advance knowledge and appropriate planning. By sharing challenges, opportunities, strengths, and weaknesses, our collective experiences could construct a foundation from which different institutions wouldbe able to create MPS accessible and relevant to the needs of their students.

In our experience, the primary obstacle was related to lack of awareness about mindfulness and the evidence base. We managed this over time through education and promotion of physician wellness, with mindfulness as one component. Continual advocacy resulted in engagement at all levels of the college and formal wellness initiatives. Due to the growing awareness of physician wellness/mindfulness, we do not feel this will be a current obstacle, at least not to the same degree.

Institutional support and logistics of implementation was and continues to be challenging. This includes communication at all levels, scheduling, room availability, access and layout, lack of curricular time, financial support, sustainable access, and support for a certified mindfulness facilitator. We provide the following recommendations to encourage discussion and collaboration in establishing MPS in medical schools.

## 1. Institutional support and student engagement is crucial to implement and sustain MPS

Stakeholder engagement and commitment should be clear from the very beginning. Their support is crucial to developing a sustainable program to address logistics including optimum use of existing resources.^11^ Engage a wide diversity of personnel including but not limited to university regulatory authorities, curriculum committees, student wellness offices, financial offices, schedulers, faculty, college administration, medical student insurance, as well as the student medical society and all wellness committees.^6^^-^^8^^,^^11^ Do not assume that the personnel, offices, and departments will communicate this information to each other.

Ideally, the college would already have a student wellness office, student society wellness committee, and wellness programming within which MPS may be offered.^12^ A certified mindfulness facilitator and champion and a small group of committed faculty and students would also facilitate implementation.^12^^,^^14^ If starting from scratch, without wellness curricula, we recommend approaching key influencers who can champion the program and equip them with current evidence relevant to the CanMEDS 2015 competencies.^13^^,^^14^ The Canadian Medical Association, Canadian Federation of Medical Students and provincial physician support programs can provide further support. The student council or a student-led wellness group^12^ could disseminate information and promote engagement via social media and speaker sessions.

We were grateful that our provincial medical association provided funding and other resources for many of these sessions. Financial remuneration and logistical support for the facilitator must be taken into consideration for sustainability.

## 2.Build mindfulness education into the curriculum

Ideally, the curriculum already contains wellness with credit and protected time.^12^ Our students reported a “lack of time” and feeling hyper-scheduled as two of their biggest stressors, which reflects the research in the literature.^1^^,^^3^^,^^6^^-^^10^ Without protected time, our students reported a feeling of guilt, pressure, and inner conflict on whether to attend the optional MPS or not. Outside the curriculum, students might not recognize the relevance and importance of mindful practice, and therefore may not prioritize attendance.^5^

If you must schedule MPS outside normal hours in the curriculum, it is not clear which time of day is most suitable.^15^^,^^18^ In the morning, students may feel the need to squeeze in extra sleep or studying. Lunch hours may be filled with student group activities, speakers, and meetings. At the end of the day, students may feel exhausted and may choose to rest, eat, exercise, or study.^6^^-^^10^

We recommend at least one mandatory large group lecture prior to MPS so that pre-clerkship students can be educated on the neuroscience and evidence of mindful practice. In our experience, a mandatory lecture enhances subsequent recruitment into MPS. In 2014, we mandated attendance to such a lecture. Sixty-nine percent showed interest in committing to at least six MPS. In 2015, we presented the info during an optional lecture with lower attendance. Even though the medical school administrators later gave all students a chance to sign up, only 54% the class showed interest in MPS.^6^^-^^10^

## 3. Offer MPS as a selective rather than a mandatory activity

In our student body, students demonstrated different levels of interest and engagement to MPS. Those who expressed interest in mindfulness appeared to benefit more than students who did not express interest.^6^^-^^10^ There are anecdotes of how uninterested students in other institutions forced into mandatory sessions have disrupted or diluted the experience for others.^16^

We were surprised by the high demand and attendance at optional sessions during examination periods, one of the busiest (and most stressful) times of the year.

## 4. Choose a facilitator with certification and clinical experience

It is essential that the facilitator has:

- Certification (e.g. Mindfulness Based Stress Reduction (MBSR)®, Mindful Practice®)
- Clinical experience. Facilitators with a health care background may relate better to the students, and frame mindfulness as a life and a clinical skill.
- A personal mindfulness practice.^17^

Exploration and guidance should be “trauma-informed” as difficult emotions and thoughts may surface during mindfulness.^4^ Administrators should clearly identify and facilitate access to mental health resources (e.g. student wellness offices, provincial health associations).

## 5. Begin with an introductory 90-minute workshop, mandatory for any students who wish to access future MPS

When we first introduced MPS in 2010, new students joined at random throughout the year. Every session, the basic principles had to be re-introduced for the benefit of the new students. This repetition limited the time available for practice and skill development.

In 2015, we introduced a foundational workshop, so that all participants understood the basic practices. The facilitator could now discuss different mindfulness concepts at subsequent sessions, deepening their skills, regardless of their frequency of attendance.^6^^-^^8^

## 6. Maintain a consistent schedule of time, day, and location

Routine is important for a regular mindful practice and neuroplastic change, and can be disrupted by long breaks between practice sessions.^4^ We found that the benefits of resiliency, coping, self-esteem subsided in our one-year follow-up to MPS.^6^^-^^10^

Consistent scheduling and dedicated space is important,^4^^,^^14^^,^^18^ and yet, was our most challenging aspect of providing MPS. We found that an unpredictable schedule and locations lowered attendance and added stress.^6^^-^^8^^,^^16^

We recommend collecting participant names and contact information. Use of sign-up sheets prior to/during the sessions assists in determining whether the students have attended all the sessions, the ability to contact them if there are any concerns or follow-up required and documents demand if further resources and funding are required.^18^

## 7. Room &equipment: Seek comfort and easy accessibility

MPS requires little set-up other than a comfortable space.^16^^,^^18^ We recommend a quiet uncluttered space. Classrooms with chairs and tables can restrict students from entering supine poses. If the facilitator and/or students spend time rearranging furniture at the beginning/end of the session, it reduces the time available for practice. A carpeted floor, an open yet private space, natural light and access to yoga mats, meditation benches and cushions promote comfort in supine and sitting positions as well as sense of security and safety. We found it best to make mats readily available with a storage unit nearby for quick set-up/take-down. It is cumbersome for students to bring their own mats.^6^^-^^8^

We chose a maximum group size of 20 participants to accommodate the space available, so that they could easily sit or lay down in a circle format. In our experience, the smaller group size promotes the sharing of personal experiences and connection. If the entire class or all interested students participate at once, potential obstacles are room size, and less time to create a safe and supportive atmosphere.^16^

In our experience, the issue of student disability insurance limited “physical activity” in the health sciences building. Mindful movement was considered an activity, and thus, MPS initially had to be booked far from the medical school in buildings that “allowed” for activity. Education, advocacy, and engagement to and for the institutions is critical for booking a room within a reasonable distance so that students do not have to run between different buildings as they are already strained for time.^6^^-^^8^^,^^12^

## 8. Base MPS content on evidence-based mindfulness courses

Our MPS are based on MBSR® and Mindful Practice®, which includes mindfulness, narrative medicine, and appreciative inquiry. This basis would allow for evidence-based facilitation and research, especially relevant in academic institutions.^19^

## 9. Other notable observations:^6^^-^^8^

- Students really seemed to connect with the “CT scan” analogy during the body scan and embraced the concepts of connecting to body and breath to anchor themselves in present moment after learning the neuroscience behind narrative and experiential circuits in the brain.
- The module on “suffering and compassion” was well received as discussion of “inner critic” and imposter syndrome allowed students to access the universal kindness meditations. We have not gathered any data that determines which modules or sessions were better received or seemed to have a greater positive effect.
- In a typical MPS, one would expect about 45 min of practice and 15 min on inquiry^1^^,^^4^^,^^18^ and discussing how it can be applied to medical practice. In our experience, students frequently stayed up to 30 minutes longer, time permitting, to share personal experiences and discuss issues, challenges, and applications with mindful practice. The sense of safety and community enhanced the experience for all.

## Closing remarks

Institutional support is crucial in implementation and sustainability of wellness and mindfulness initiatives. We recommend that all pre-clerkship students have a basic understanding of mindfulness as a clinical and a life skill. This could be introduced during a lecture, which may enhance recruitment for MPS. MPS should be built into the curriculum as a selective rather than a mandatory activity. A foundational workshop should be offered to students interested in future sessions. MPS delivered outside the curriculum and off-site may results in lower attendance. MPS offers an exciting evidence-based opportunity for experiential learning. We acknowledge that these recommendations may not be applicable to all medical schools, given the variation in curriculum, location, human resources, and other factors. These recommendation on how to get a grip may change in light of future research, resources and supports.
